# Addressing Health-Related Social Needs and Mental Health Needs in the Neonatal Intensive Care Unit: Exploring Challenges and the Potential of Technology

**DOI:** 10.3390/ijerph20247161

**Published:** 2023-12-09

**Authors:** Eline van de Kamp, Jasmin Ma, Nagendra Monangi, Fuchiang Rich Tsui, Shilpa G. Jani, Jae H. Kim, Robert S. Kahn, C. Jason Wang

**Affiliations:** 1 Athena Institute, Faculty of Science, Vrije Universiteit Amsterdam, 1081 HV Amsterdam, The Netherlands; e.d.vande.kamp@student.vu.nl; 2 Center for Policy, Outcomes, and Prevention, Department of Pediatrics, Stanford University School of Medicine, Stanford, CA 94305, USA; jyma@stanford.edu (J.M.); sgjani@stanford.edu (S.G.J.); 3 Division of Neonatology, Perinatal Institute, Cincinnati Children’s Hospital Medical Center, Cincinnati, OH 45229, USA; nagendra.monangi@cchmc.org (N.M.); jae.kim@cchmc.org (J.H.K.); 4 Department of Pediatrics, University of Cincinnati College of Medicine, Cincinnati, OH 45267, USA; robert.kahn@cchmc.org; 5 Tsui Laboratory, Department of Biomedical and Health Informatics, Children’s Hospital of Philadelphia, Philadelphia, PA 19146, USA; tsuif@chop.edu; 6 Department of Anesthesiology and Critical Care Medicine, Perelman School of Medicine, University of Pennsylvania, Philadelphia, PA 19104, USA; 7 Michael Fisher Child Health Equity Center, Cincinnati Children’s Hospital Medical Center, Cincinnati, OH 45229, USA; 8 Department of Pediatrics and Department of Health Policy, Stanford University School of Medicine, Stanford, CA 94305, USA

**Keywords:** social determinants of health, health-related social needs, parental mental health, neonatal intensive care unit, mobile health technology, social work, infant health, infant development

## Abstract

Unaddressed health-related social needs (HRSNs) and parental mental health needs in an infant’s environment can negatively affect their health outcomes. This study examines the challenges and potential technological solutions for addressing these needs in the neonatal intensive care unit (NICU) setting and beyond. In all, 22 semistructured interviews were conducted with members of the NICU care team and other relevant stakeholders, based on an interpretive description approach. The participants were selected from three safety net hospitals in the U.S. with level IV NICUs. The challenges identified include navigating the multitude of burdens families in the NICU experience, resource constraints within and beyond the health system, a lack of streamlined or consistent processes, no closed-loop referrals to track status and outcomes, and gaps in support postdischarge. Opportunities for leveraging technology to facilitate screening and referral include automating screening, initiating risk-based referrals, using remote check-ins, facilitating resource navigation, tracking referrals, and providing language support. However, technological implementations should avoid perpetuating disparities and consider potential privacy or data-sharing concerns. Although advances in technological health tools alone cannot address all the challenges, they have the potential to offer dynamic tools to support the healthcare setting in identifying and addressing the unique needs and circumstances of each family in the NICU.

## 1. Introduction

Health-related social needs (HRSNs) encompass adverse socioeconomic factors that can affect health outcomes, including issues such as food insecurity, housing instability, limited access to transportation, and unemployment. Unaddressed HRSNs can lead to decreased access to and utilization of needed healthcare services, which in turn negatively impact health outcomes [1,2,3,4,5,6].

The admission of infants to the neonatal intensive care unit (NICU) is known to be associated with HRSNs and adverse parental mental health. In particular, income, race/ethnicity, and insurance status have been associated with preterm birth, neonatal abstinence syndrome, and readmissions [7,8,9,10]. Unaddressed HRSNs in the early stages of life also increase the likelihood of the early onset of chronic health conditions, developmental delays, and poorer academic performances [11,12,13]. Consequently, infants admitted to the NICU are disproportionately affected by HRSNs and adverse health outcomes, which may have lifelong implications [14,15,16]. In addition, around 40% of parents with infants admitted to the NICU experience clinically significant symptoms of depression or anxiety [17], as well as high rates of posttraumatic stress disorder after NICU discharge [18]. Untreated mental health disorders in parents are further associated with adverse infant health outcomes and decreased parent–infant bonding [19,20,21]. Thus, addressing the HRSNs and mental health of families with infants in the NICU may have a significant impact on infant health outcomes.

Although the American Academy of Pediatrics advocates for universal HRSN screening within the pediatric setting and encourages collaborations with community-based organizations [22], the current implementation of these practices in NICUs across the U.S. remains sparse. Only about 1 in 4 NICUs in the U.S. currently have a standardized HRSN screening process [23,24]. While most multidisciplinary NICU teams include social workers, their wide range of responsibilities to help families cope in the NICU may limit their bandwidth to thoroughly screen for and address HRSNs or parental mental health needs [16]. Other known challenges in addressing these needs in the NICU include perceptions of a lack of validated and/or standardized screening tools, inadequate training, limited resources to address identified needs, and difficulties integrating practices into existing workflows [16,23,25,26,27]. Caregiver-related factors can pose additional challenges to interventions, such as the competing demands of caring for a medically complex child, difficulties navigating resources and applying for benefits, language barriers, and concerns about stigma or discrimination [27,28,29].

The utilization of advanced digital tools may help facilitate the process of identifying and addressing HRSNs and parental mental health needs to the benefit of healthcare staff, patients, and caregivers. For example, there have been attempts to apply natural language processing to extract and categorize HRSNs from unstructured clinical notes to improve documentation [30,31]. Previous studies have also found that electronic screeners and resource directories can reduce the burden on staff, mitigate potential biases, and improve efficiency, screening rates, and resource connection [27,28,32].

While the potential of using digital tools to improve community resource referrals has been explored [33], the unique conditions and challenges in the NICU require further exploration to understand how technology can aid interventions for complex needs. To bridge this gap, this study aims to (1) explore the current processes and challenges encountered by the NICU care team in identifying and addressing HRSNs or parental mental health needs and (2) identify ideas and considerations for using technology to aid HRSN and mental health interventions.

## 2. Materials and Methods

### 2.1. Study Design

This study adopted an interpretive description (ID) approach utilizing purposeful sampling, concurrent data collection and analysis, and inductive coding [34]. The ID methodology was selected for its exploratory nature, allowing a deeper understanding of a phenomenon within its context. It aims to generate relevant knowledge that is directly applicable to clinical settings [35]. Semistructured stakeholder interviews were conducted to achieve three main objectives: (1) explore the existing screening and referral processes for addressing the HRSNs and mental health needs of families with infants in the NICU; (2) investigate the challenges experienced by the NICU care team in these processes; and (3) assess the potential of technology, as perceived by the NICU care team and other relevant stakeholders, in addressing these challenges. The Institutional Review Board at Stanford University determined this study (Protocol #69405) to be exempt from Human Subject Research.

### 2.2. Research Setting

Interviews were conducted at three sites: the Children’s Hospital of Philadelphia (CHOP), PA; the Cincinnati Children’s Hospital Medical Center (CCHMC), OH; and the Lucile Packard Children’s Hospital (LPCH), CA. All three hospitals are safety net providers with level IV NICUs in their respective regions. As safety net providers, these hospitals provide services regardless of insurance status or ability to pay. Consequently, they serve a substantial number of uninsured and low-income populations, making them strategic places to understand the impact of HRSNs on families with NICU infants. As level IV NICUs provide care to infants with the most severe conditions, challenges at the selected sites are likely to reflect the needs of the most medically and socially vulnerable populations. The NICUs at CHOP, CCHMC, and LPCH consist of 95, 93, and 40 beds, respectively, and provide family-centered care, considering parents to be essential to the health and well-being of their infants. Parents are actively encouraged to visit the NICU and participate in the care of their baby. The interviews were first conducted at CHOP, and the findings were subsequently cross-validated at CCHMC and LPCH to identify common themes.

### 2.3. Participants

We approached staff at each of the three sites, who assisted in identifying individuals with roles directly involved in screening or providing services for HRSNs or mental health to families of infants admitted to the NICU. At the end of each interview, the participants were asked to recommend others who might be able to provide additional insights. Interview participants consisted of members of the NICU care team and key stakeholders, such as the high-risk infant follow-up team, physician-leaders engaged in policy implementation related to addressing HRSNs and establishing community partnerships, and representatives of community organizations.

### 2.4. Data Collection

Initial prompt questions were generated for each role, exploring the current screening and referral processes for HRSNs and mental health needs, challenges in the existing processes, and the perceived potential or concerns about the use of technology to facilitate these processes (see Appendix A). The questions were designed to be open-ended, and specific items were iteratively added or adapted to account for insights from previous interviews. All the interviews were conducted virtually, recorded with consent, and transcribed verbatim.

### 2.5. Data Analysis

Thematic analysis of the transcripts was performed using ATLAS.ti Windows (Version 23.2.3) and ATLAS.ti Web (Version 5.11.1) [36], employing an inductive coding approach. Codes were identified, reviewed, and categorized to generate common themes. To enhance data validity, two researchers independently analyzed the data to limit bias and to help establish intercoder reliability (investigator triangulation), and the findings underwent review by established experts in the field (expert triangulation) [37]. The interviews were conducted until thematic saturation was achieved and no new themes emerged upon analyzing additional interviews.

## 3. Results

In total, 22 individuals were interviewed, including 12 participants associated with CHOP, 5 associated with CCHMC, and 5 associated with LPCH. The roles and range in years of experience of the interview participants are detailed in Table 1. The analysis of the interview data revealed five overarching themes encompassing the challenges in addressing HRSNs and parental mental health needs in the NICU. Table 2 presents the major themes and associated quotes from participants.

### 3.1. Screening

The participants mentioned how certain needs are challenging to address in the NICU setting, specifically mental health and housing issues. Limited resources, long wait times, eligibility constraints, and limited insurance coverage often hinder families from accessing the necessary support. The lack of resources raises a dilemma: whether to screen for needs if those needs cannot be appropriately addressed. Those who advocated for screening only when meaningful follow-up support was available argued that it was the fairest and most ethical approach. They believed that uncovering needs without meaningful action could lead to stigmatization and harm to the families. On the other hand, participants in favor of screening for needs, regardless of resource availability, argued that even if social workers could not address the needs directly, they could still provide strategies or initial steps for the families or adjust their care. One social worker commented that such screening allowed them “to better understand that person and where that person is coming from”. The participants also emphasized that social workers and care teams must be aware of a family’s housing situation to ensure a safe discharge from the NICU. Additionally, being aware of families’ needs has broader implications for informing policy and partnerships to address these needs. However, most participants did acknowledge that it is a challenge to screen for needs without the resources to respond to them adequately.

None of the institutions had a hospital-wide standardized screening protocol for HRSNs or mental health needs. This is mainly due to concerns about privacy, stigma, capacity, and lack of resources, resulting in significant discrepancies in screening approaches across different hospital settings. Also, all the needs were not always assessed during the initial psychosocial assessment to avoid information overload. Instead, topics related to complex infant care and urgent needs were given priority. Additionally, needs may be documented only in the parent or infant’s electronic health record (EHR), resulting in a disjointed documentation of needs. For example, HRSNs and mental health needs identified during prenatal care are often not included in the infant’s EHR. Within the NICU, variability exists in what parental needs are recorded in the infant’s EHR and the level of detail included. Social workers and psychologists are especially cautious when entering information about a caregiver’s mental health needs into an infant’s EHR. The current approaches lead to significant lapses in the transfer of information across the healthcare system taking care of maternal–infant dyads during this critical phase (e.g., obstetrics, NICU, psychiatry, high-risk infant follow-up, and primary care). Consequently, when families encounter other parts of the healthcare system, redundant screening efforts may occur, resulting in screening fatigue and maldistribution of resources that are duplicative for some and lacking or ineffective for others.

The timing of screening for HRSNs and mental health as well as the selection of appropriate screening tools pose challenges in the context of the NICU. The optimal time for screening can be complex, as needs are dynamic rather than static, and families may already be overwhelmed by the hospitalization of their infant. As described by one social worker, “parents are overburdened with a great amount of information… when their primary concern is just the safety of their baby.” In terms of parental mental health screening, there are concerns that the existing screening tools may not be suitable, given differing baseline levels as a result of the NICU experience. One NICU psychologist noted that caregivers in the NICU are expected to have “a certain level of anxiety, a little stress, [and] depression”. While increasing screening for and monitoring of evolving needs could be beneficial, this approach also raises concerns about additional burdens on social workers and families in the NICU. Participants also had different perspectives on the utilization of screening tools versus direct inquiry, where families are simply asked whether they have any needs or require assistance. While the latter approach could potentially mitigate the unintended consequences of comprehensive screenings, such as fear of stigma and judgment, validated tools allow for a more standardized approach.

### 3.2. Referrals and Care Coordination

A common issue is that no automated systems are in place to track referrals made, and closed-loop referrals are often lacking. As a community health program manager stated, “we refer families to services all the time and have no idea what happened”. In most cases, social workers depend on check-in conversations at the bedside to learn whether the family followed up with referrals, qualified for services, or received resources. There are some exceptions where social workers would contact an organization to receive updates or have established collaborations that involve frequent data exchanges between both sides. Nevertheless, maintaining these closed-loop communications is difficult as many different organizations are involved, and putting more (administrative) burden on these organizations and the social workers should be avoided.

Moreover, not all families can be consistently present in person—particularly on weekdays during work hours—for staff to effectively identify needs or follow-up. Families could face multiple barriers that may prevent frequent visits, such as juggling multiple jobs, caring for their other children, or long travel distances/lack of transportation. Consequently, these families could miss out on important check-ins and have needs that are compounded by their infant’s hospitalization.

The transition to home from the NICU and the postdischarge period can be highly challenging for families, and they often need additional support and assistance in navigating resources. This situation presents a challenge for NICU social workers. On the one hand, they may strive to offer as much assistance as possible and, on the other hand, encourage or rely on families to seek out resources more independently. Per one social worker, after discharge from the NICU, “there’s usually a gap because a lot of the time social workers and providers aren’t going to make contact with the family until their first follow-up appointment.” Moreover, the level of support provided in the pediatric outpatient setting, particularly for parental mental health needs, is often not as comprehensive as what was provided in the NICU. Families need to assume more responsibility to seek and access resources themselves, which can be challenging during this transitional phase. In particular, non-English-speaking families encounter many difficulties in finding and applying for resources independently.

### 3.3. Use of Technology

**Figure 1:**
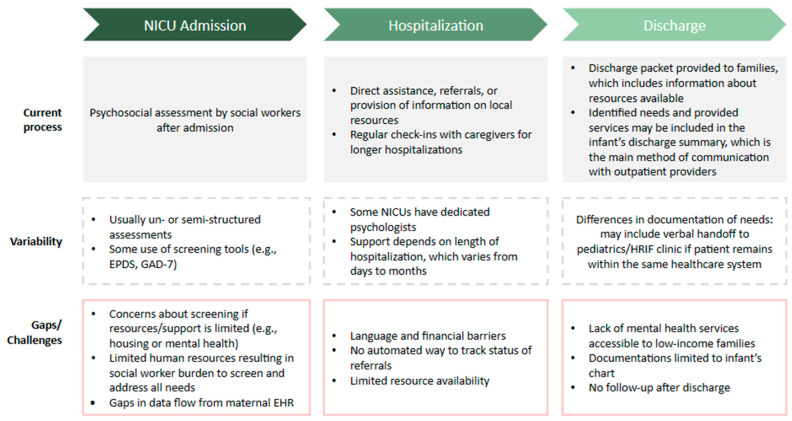
Current process and barriers. HRIF: high-risk infant follow-up.

The current process of addressing HRSNs and parental mental health needs in the NICUs across sites is shown in Figure 1, along with the variation in practices among the sites and the common challenges identified by participants.

In relation to the challenges, many participants highlighted the potential benefits of technology in increasing screening rates, improving triage efficiency, and reducing the burden on providers. However, this requires the identification of appropriate validated tools and methods that facilitate timely support for higher-risk families. Automated referral systems and resource mapping have the potential to enhance families’ awareness and access to eligible resources and also aid in appointment scheduling. Digital platforms further offer opportunities for accessing credible health education, providing hospital-specific information, fostering peer-to-peer support, and facilitating communication between providers and patients—particularly in providing needed support after NICU discharge. Finally, some participants indicated that technology could help reduce language barriers overall, particularly for resource provision.

The participants also discussed the impact of increasing reliance on technology, particularly for underresourced populations. Some were concerned about the possibility of perpetuating disparities for those without smartphones or internet access. For mental health services, some providers commented on a potential increase in access with the availability of virtual consultations. Lastly, considerations about privacy and data-sharing were mentioned, particularly considering how and what patient data should be reasonably shared with other providers and community organizations if information-sharing becomes more automated. Comments included the need to ensure that automated referrals do not reduce resource eligibility and that documentation cannot be used against patients. Usability must also be considered to ensure that designs improve efficiency and do not add to the burden of families.

## 4. Discussion

Given the importance of addressing HRSNs and parental mental health needs, it is essential to continue improving screening and referral practices, particularly in the NICU setting where families tend to have a higher risk of unmet needs, and any existing unmet needs may be exacerbated during a NICU stay [28,38]. Moreover, as preterm birth rates and subsequent NICU hospitalizations are more prevalent among low-income populations, enhancing screening and referral practices in the NICU becomes crucial for effectively addressing the unmet needs of these vulnerable groups [38].

The existing literature brings attention to the importance of HRSN and parental mental health screening in the NICU setting and provides assessments of current and possible approaches. Building upon this, our study identified specific challenges with addressing these needs in the NICU and continuing after discharge, as perceived by key stakeholders of the care team. As similarly voiced by the participants in our study, one perspective comments on the compounding effects of HRSNs on families with an infant in the NICU and emphasizes the need for clinicians to support families after discharge [39]. Also, in concordance with our findings, other studies have found that HRSNs were not consistently assessed in NICUs across the U.S. despite the fact that most clinical leaders believed it to be beneficial [23,24]. However, efforts have been made to demonstrate that standardized screening in the NICU is possible [16], and our study contributes by adding considerations for the use of digital tools in this process.

Leveraging technology in the screening and referral process can potentially address the unintended consequences of direct face-to-face screening. Firstly, electronic screening can be completed by families in a comfortable and private environment, mitigating the barriers and stigma associated with discussing sensitive issues in person. Secondly, electronic screening of a family’s unmet needs may reduce the burden on social workers and care providers, allowing them to effectively identify areas of concern that require timely attention or intervention. Thirdly, integrating electronic screeners directly with the EHR can further improve the documentation of HRSNs and mental health needs, ensuring that identified needs are communicated effectively and seamlessly across different healthcare settings. Fourthly, resource availability can be improved by streamlining and standardizing the referral processes for unmet HRSNs and parental mental health needs. Implementing an automatic triaging system facilitates seamless connections between families and resources, making the support process more efficient. For example, participants expressed the importance of having a tiered approach for mental health risk assessments. Screening through technology allows for the automatic flagging of the risk-based positive results, timely referral to a social worker, and if appropriate, to a psychologist based on the severity of the needs. Finally, implementing a closed-loop system holds significant potential in efficiently tracking referrals, ensuring that families receive the necessary support and offering valuable insights into the challenges faced by families when following up with referrals.

Technology may also mitigate the challenges faced by families who cannot regularly be at the bedside in the NICU. Through technology, more regular check-ins can occur, gaining insights into the developing needs and the state of referrals, even when physical presence is not possible. Offering support groups virtually could help lower barriers to participation. This way, families can connect, share experiences, and provide support, even if they cannot attend in-person meetings. Additionally, technology can facilitate the connection between families and their infants in the NICU through video feeds and other virtual communication tools.

Technology could provide a comprehensive overview of the available resources to support families in navigating resources independently during and after the NICU hospitalization. By offering families access to this overview through a user-friendly platform, they can be enabled to explore resources at their convenience while also reducing the likelihood of information getting lost, as might happen with a paper-based list. Furthermore, the overview could be enhanced by making it more organized and personalized. For example, the platform could indicate the eligibility criteria for the resources and whether they will need prior authorization from or be covered by their insurance. This can mitigate the burden and confusion that accompanies navigating resources and provides a smoother transition out of the NICU.

Other potentials of technology include offering screenings and guidance for resources in multiple languages and providing education resources related to the NICU stay and the potential development of HRSNs and mental health needs during this period. By presenting validated information on a platform, families can access reliable and trustworthy content instead of needing to search for it themselves and possibly finding inaccurate information online.

Despite all these potential advantages of leveraging technology, certain challenges remain regarding where and how HRSNs and parental mental health needs should be documented and the willingness of families to disclose needs. Similarly, improved screening strategies must also account for resource availability, patient priority, and readiness. Although closed-loop referrals have been successfully implemented on a smaller scale, integrating them into the wider referral system presents challenges that need careful consideration. One of the primary concerns is data sharing and privacy issues, as the exchange of information between prenatal clinics, NICUs, primary care clinics, and community-based organizations requires the careful handling of sensitive information. In addition, managing the data and effectively coordinating the referral process may add significant administrative burdens at all levels. To overcome these challenges, further research is needed to identify optimal strategies for implementing a closed-loop system in the NICU setting, including providing different levels of access to information granted by the user.

The limited availability of certain resources emerged as a significant challenge during the interviews, which is well documented in the literature as well [40,41]. Technology cannot solve these underlying issues. However, it could facilitate a triage approach, where only high-risk individuals are seen immediately by a specialist, such as those experiencing a mental health crisis. Screening can enable early identification and intervention before situations worsen, reducing the number of individuals requiring urgent specialized care [42]. Furthermore, screening for difficult-to-address needs can highlight service gaps; drive advocacy for additional staffing resources, such as psychologists in the NICU; and foster stronger partnerships with community-based organizations with necessary resources, which might help mitigate resource availability issues in the future [40].

When addressing the barriers faced in the NICU in addressing HRSNs and mental health needs by leveraging technology, significant considerations should be made to ensure the effectiveness and equity of the approach. This includes ensuring that the families in greatest need are reached. Although the smartphone ownership rate among U.S. adults reached 85% in 2021, the proportion lowers to three in four for those with a household income under $30,000 or who only completed high school or less [43]. Factors such as tech savviness, health literacy, and language should also be considered to avoid unintentionally perpetuating disparities or leaving people out.

This study includes some limitations. The iterative approach to data collection utilized in this study allowed for a focused exploration of relevant areas, enhanced the reliability of the results, and facilitated the identification of gaps and emerging themes. However, because the three sites included in this study were all major hospitals with level IV NICUs that had some system in place to address HRSNs and mental health needs, smaller hospitals with fewer resources may face additional challenges that are not described in this study. As the level of access to risk-appropriate perinatal care contributes to disparities in neonatal outcomes [44], further studies are needed to better understand the perinatal experience of high-risk populations at lower-level hospitals and the challenges in providing perinatal care at these sites. In addition, this study only included the perspectives of care providers and researchers. Perspectives from the caregivers of infants in the NICU may shed additional insights on barriers to care, particularly as digital tools become more prevalent.

## 5. Conclusions

Addressing adverse infant outcomes resulting from HRSNs and mental health needs within families is of crucial importance due to their potential lifelong implications. The NICU setting offers a unique opportunity to reach these families and address their needs. Therefore, it is important to support the NICU care team in identifying and addressing these needs while finding ways to mitigate their challenges. The growing recognition of the importance of addressing HRSNs and mental health needs to improve health outcomes has led to significant advances in clinical approaches in recent years. However, the current approach to addressing these needs remains fragmented and lacks a systematic framework for the implementation of effective screening and referral practices.

Technology can serve as a promising tool in supporting the progress toward addressing unmet needs and improving infant health outcomes. To fully realize the potential benefits of technological interventions within the NICU setting, future research needs to investigate their effectiveness and ensure a family-centered approach. The perspectives of the population intended to benefit from these interventions (e.g., parents, guardians, and community partners) should be explored. Engaging the community, particularly the most vulnerable and disadvantaged groups, will provide insight into how the use of technology can be prevented from being seen as burdensome. Additionally, the feasibility and efficiency should be thoroughly investigated.

## Figures and Tables

**Table 1:** Participant characteristics.

Institutions		
CHOP ^1^ (*n* = 12)	CCHMC ^2^ (*n* = 5)	LPCH ^3^ (*n* = 5)
Roles/Job descriptions (years in practice) *		
NICU care team: Social worker (1–24) Psychologist (1–14) Neonatologist (8–11) Care manager (4–6) Other stakeholders: Physician-researcher (8–19) High-risk infant follow-up physician (5–7) Program manager (community health, public health, maternal care) (12–16) Clinical research/resource mapping coordinator (1–3)		

^1^ Children’s Hospital of Philadelphia; ^2^ Cincinnati Children’s Hospital Medical Center; ^3^ Lucile Packard Children’s Hospital at Stanford. * Some participants hold more than one role.

**Table 2:** Major themes and quotes from semistructured interviews.

Major Themes	Quotes
Screening with meaningful follow-up	“We screen for everything even if we don’t have a perfect solution because even if we can’t do anything about it, we are at least able to better understand that person and where that person is coming from. And maybe we can’t find them stable housing, but we can help them with [...] other things that might make the needs that they have less stressful.” (NICU social worker)
Standardizing and streamlining the processes	“Needs or issues that arose within a NICU hospitalization [are documented] into the paperwork that families get discharged with. That hopefully then makes it to the primary care provider. [...] But you can already start to see that if you are dependent on documentation and a paper trail for families’ issues, there’s obviously gaps that emerge in terms of what we’ve discovered or worked on during the NICU and how that makes it into a primary care provider’s ability to continue to be aware of that.” (Physician-researcher) “Some of the screening measures I think aren’t validated to a NICU population [...] Certainly, parents who have a baby, there’s a certain level of anxiety, a little stress, depression that might come with that. You know, baby blues plus postpartum depression. But in the NICU, I mean, it’s a given, right? [...] Are you sleeping? Well, of course NICU parents are not sleeping, right? They’re concerned about their baby.” (NICU psychologist)
Burdens resulting from the NICU experience	“What I’ve come to recognize as our parents are overburdened with a great amount of information, and their brains are in crisis and are in fight or flight so to expect to kind of go in initially asking all of these heavy questions, when their primary concern is just the safety of their baby. Or just kind of asking them what their primary concern is, helps with the Maslow hierarchy of needs, like whatever they feel is most important in this time is kind of the approach we need to have versus kind of our own plan that we would like to have our own agenda per se.” (NICU social worker) “Not everybody is here between 8:00 and 6:00. You know, people are working. They have families. You know, they may not be able to be here during working hours or during the weekdays. And we would miss that population. And if you think about it, those folks who are busy like that probably have a higher risk, right? Because they’re trying to juggle being at the hospital, working, other children. So you know that already puts them in the category of, you know, stressed.” (NICU psychologist) “The problem, of course, is that these families are in crisis, right? We tell them medical information and they don’t absorb it at all, like the next day you have to tell them the same thing again. And it’s just because they’re so stressed, they can’t remember it. Which is a benefit of having something digital, because then if they do find a moment when they are a little bit more calm and can deal with something they can go back to it.” (Neonatologist)
Maintaining support and closing the loop	“We refer families to services all the time and have no idea what happened, no idea if they were connected, no idea if they got the service. And one of the things we’re trying to think about is, if a family comes in and they’re food insecure, for example, they need help with food resources and we connect them for SNAP issues, they apply for SNAP, and get food from the food pantries. How do we prevent them from two months later coming back and they’re still food insecure?” (Program Manager Community Health) “There’s usually a gap because a lot of the time social workers and providers aren’t going to make contact with the family until their first follow-up appointment. So if they’re being followed by neurology but they discharge and their first neurology appointment isn’t for three months, then they’re going to go that three months without really having anybody.” (NICU social worker)
Expectations and reliance on the family	“I do try to still have some expectations of accountability for some of our families, because we can easily enable a lot sometimes and provide and provide to the point the child’s almost ready for discharge and there’s kind of this expectation of like, ‘Well, where am I gonna get my formula? Where am I going to get my diapers?’ [...] So we try to have a balance between assisting at this time of crisis for them, as well as still expecting some responsibility towards providing things for their child within reason.” (NICU social worker) “Sometimes I’m able to follow up [with the family] and know that they’ve utilized these resources. Otherwise, sometimes I just make the referrals and then it’s up to the family if they’re going to follow through with them or ultimately use them.” (NICU social worker)

## Data Availability

The data presented in this study are available on request from the corresponding author. The data are not publicly available due to privacy restrictions.

## Appendix A

**General Questions:**

- Can you describe your roles/responsibilities as a ______?
- What do you think are the main challenges to addressing HRSNs/parental mental health needs in (your role/setting)?
- How do you feel about assessing needs that you might not be able to help the family with, for example, housing?
- Do you think standardized screening for (HRSNs/mental health) should be implemented? Why or why not?
- How do you find out about the needs that the families might have? How do you coordinate or communicate identified needs to (other departments or specialties within the health system/community-based organizations)?
- How commonly are HRSN and parental mental health needs identified in (your setting)? What are some of the main HRSNs or mental health needs you see?
- How are needs documented (e.g., in the infant’s chart)?
- How are resources and support provided once needs are identified?
  o Is there any follow-up during the following visits? If so, how is that accomplished?
- Are there any challenges with families that do not have English as their first language?
- What do you think are the potential roles of technology in reducing the challenges of identifying, addressing, or coordinating the care of HRSN and parental mental health within the NICU and after discharge? Or do you have any concerns about how technology is being/may be used in this process?
- Do you know those in other roles who we might be able to talk to to learn more about NICU families’ experiences with (HRSN/parental mental health needs)?
- Is there anything we haven’t discussed that you would like to add?

**NICU Social Worker Questions:**

- What HRSN or mental health needs do you assess for when you meet families?
  o Do all social workers conduct assessments in the same way or is there variability in how this is accomplished?
  o How do you learn about any new/developing needs families might have after the initial visit?
- If you make a referral where a family has to contact a community-based organization or service themselves, how do you obtain insight into the status of the referral?
- After discharge from the NICU, do families still reach out to you for support in navigating resources?
  o Do you have contact with the social worker that will be the next point of contact for the family?
  o Are there any difficulties families face after discharge that you are aware of?
- Are there any difficulties providing information about referrals to families, and do you know of any issues families face getting connected with organizations/services?

**NICU Psychologist Questions:**

- How are parental mental health needs identified in the NICU? Does the social worker or do you screen for this?
  o Are families universally screened?
  o Are any specific tools used?
  o When is the initial screening and are there any follow-up screenings?
- How many of the NICU families do you typically meet? Are you able to meet all the families that need it?
  o If not all, how do you decide who is seen?
  o Is anything offered to families that you do not meet?
- Do you always meet with families in person or is there also a possibility for families to meet online?
- What happens at discharge? Are you still able to provide support for families or refer them to other mental health support programs?
  o Do you encounter any issues connecting families with outpatient mental health services?
- How closely do you work together with the social worker?
  o How much information is available to you about the HRSNs of a family?

**Neonatologist Questions:**

- In your role, how do you usually hear about any health-related social needs or parental mental health needs for your patients? Do you usually hear about them from other NICU staff or do families often talk about these concerns in your interactions with them as well?
- Is there a formal moment where information is exchanged within the NICU staff about the HRSN or mental health needs of a family or infant?

**Questions for Other Roles:**

- How does the support from social workers in the inpatient setting (e.g., NICU/PICU) compare to that of the outpatient setting?
- What proportion of NICU graduates are seen in the high-risk infant follow-up (HRIF) clinic?
  o What is the interval for HRIF visits?
  o What age are patients seen until or does it vary?
- Does any HRSN or parental mental health screening take place during HRIF?

## References

[1] Office of Disease Prevention and Health Promotion Social Determinants of Health—Healthy People 2030. https://health.gov/healthypeople/priority-areas/social-determinants-health.

[2] Braveman P., Egerter S., Williams D.R. (2011). The Social Determinants of Health: Coming of Age. Annu. Rev. Public Health.

[3] Alley D.E., Asomugha C.N., Conway P.H., Sanghavi D.M. (2016). Accountable Health Communities—Addressing Social Needs through Medicare and Medicaid. N. Engl. J. Med..

[4] Adepoju O.E., Liaw W., Patel N.C., Rastegar J., Ruble M., Franklin S., Renda A., Obasi E., Woodard L. (2022). Assessment of Unmet Health-Related Social Needs Among Patients With Mental Illness Enrolled in Medicare Advantage. JAMA Netw. Open.

[5] Marmot S.M. (2009). Closing the health gap in a generation: The work of the Commission on Social Determinants of Health and its recommendations. Glob. Health Promot..

[6] Marmot M.G., Bell R. (2009). Action on Health Disparities in the United States. JAMA.

[7] Meyer M.B., Kopp S.J., DeFranco E., Kelly E. (2021). 19 Social determinants of health in preterm birth of non-Hispanic black women. Am. J. Obstet. Gynecol..

[8] Hong X., Bartell T.R., Wang X. (2020). Gaining a deeper understanding of social determinants of preterm birth by integrating multi-omics data. Pediatr. Res..

[9] Decker C.M., Mahar M., Howells C.L., Ma Z.-Q., Goetz C.T., Watkins S.M. (2023). Demographics, Birth Parameters, and Social Determinants of Health Among Opioid-Exposed Mother-Infant Dyads Affected by Neonatal Abstinence Syndrome in Pennsylvania, 2018–2019. Matern. Child Health J..

[10] Hannan K.E., Hwang S.S., Bourque S.L. (2020). Readmissions among NICU graduates: Who, when and why?. Semin. Perinatol..

[11] Malat J., Oh H.J., Hamilton M.A. (2005). Poverty Experience, Race, and Child Health. Public Health Rep..

[12] Cook J.T., Frank D.A. (2008). Food Security, Poverty, and Human Development in the United States. Ann. N. Y. Acad. Sci..

[13] Wood D. (2003). Effect of Child and Family Poverty on Child Health in the United States. Pediatrics.

[14] Shonkoff J.P., Garner A.S., Committee on Psychosocial Aspects of Child and Family Health, Committee on Early Childhood, Adoption, and Dependent Care, Section on Developmental and Behavioral Pediatrics (2012). The Lifelong Effects of Early Childhood Adversity and Toxic Stress. Pediatrics.

[15] Grunberg V.A., Geller P.A., Bonacquisti A., Patterson C.A. (2018). NICU infant health severity and family outcomes: A systematic review of assessments and findings in psychosocial research. J. Perinatol..

[16] Cordova-Ramos E.G., Jain C., Torrice V., McGean M., de la Vega P.B., Burke J., Stickney D., Vinci R.J., Drainoni M.-L., Parker M.G. (2023). Implementing Social Risk Screening and Referral to Resources in the NICU. Pediatrics.

[17] Grunberg V.A., Geller P.A., Hoffman C., Njoroge W., Ahmed A., Patterson C.A. (2021). Parental mental health screening in the NICU: A psychosocial team initiative. J. Perinatol..

[18] Schecter R., Pham T., Hua A., Spinazzola R., Sonnenklar J., Li D., Papaioannou H., Milanaik R. (2019). Prevalence and Longevity of PTSD Symptoms Among Parents of NICU Infants Analyzed Across Gestational Age Categories. Clin. Pediatr..

[19] Hoffman C., Dunn D.M., Njoroge W.F.M. (2017). Impact of Postpartum Mental Illness Upon Infant Development. Curr. Psychiatry Rep..

[20] Carter A.S., Garrity-Rokous F.E., Chazan-Cohen R., Little C., Briggs-Gowan M.J. (2001). Maternal Depression and Comorbidity: Predicting Early Parenting, Attachment Security, and Toddler Social-Emotional Problems and Competencies. J. Am. Acad. Child Adolesc. Psychiatry.

[21] Zelkowitz P., Na S., Wang T., Bardin C., Papageorgiou A. (2011). Early maternal anxiety predicts cognitive and behavioural outcomes of VLBW children at 24 months corrected age. Acta Paediatr..

[22] Gitterman B.A., Flanagan P.J., Cotton W.H., Dilley K.J., Duffee J.H., Green A.E., Keane V.A., Krugman S.D., Linton J.M., Council on Community Pediatrics (2016). Poverty and Child Health in the United States. Pediatrics.

[23] Cordova-Ramos E.G., Kerr S., Heeren T., Drainoni M.-L., Garg A., Parker M.G. (2022). National Prevalence of Social Determinants of Health Screening Among US Neonatal Care Units. Hosp. Pediatr..

[24] Parker M.G., Garg A., Brochier A., Rhein L.M., Forbes E.S., Klawetter S., Drainoni M.-L. (2020). Approaches to addressing social determinants of health in the NICU: A mixed methods study. J. Perinatol..

[25] Schwartz B., Herrmann L.E., Librizzi J., Gayle T., Waloff K., Walsh H., Rucker A., Herrera N., Bhansali P. (2020). Screening for Social Determinants of Health in Hospitalized Children. Hosp. Pediatr..

[26] Lake K.J., Boyd M.A., Smithers L., Howard N.J., Dawson A.P. (2022). Exploring the readiness of senior doctors and nurses to assess and address patients’ social needs in the hospital setting. BMC Health Serv. Res..

[27] Berry C., Paul M., Massar R., Marcello R.K., Krauskopf M. (2020). Social Needs Screening and Referral Program at a Large US Public Hospital System, 2017. Am. J. Public Health.

[28] Vasan A., Darko O., Fortin K., Scribano P.V., Kenyon C.C. (2021). Community Resource Connection for Pediatric Caregivers With Unmet Social Needs: A Qualitative Study. Acad. Pediatr..

[29] Steeves-Reece A.L., Totten A.M., Broadwell K.D., Richardson D.M., Nicolaidis C., Davis M.M. (2022). Social Needs Resource Connections: A Systematic Review of Barriers, Facilitators, and Evaluation. Am. J. Prev. Med..

[30] Richie R., Ruiz V.M., Han S., Shi L., Tsui F. (2023). Extracting social determinants of health events with transformer-based multitask, multilabel named entity recognition. J. Am. Med. Inform. Assoc..

[31] Han S., Zhang R.F., Shi L., Richie R., Liu H., Tseng A., Quan W., Ryan N., Brent D., Tsui F.R. (2022). Classifying social determinants of health from unstructured electronic health records using deep learning-based natural language processing. J. Biomed. Inform..

[32] Fortin K., Vasan A., Wilson-Hall C.L., Brooks E., Rubin D., Scribano P.V. (2021). Using Quality Improvement and Technology to Improve Social Supports for Hospitalized Children. Hosp. Pediatr..

[33] Iott B.E., Eddy C., Casanova C., Veinot T.C. (2020). More than a Database: Understanding Community Resource Referrals within a Socio-Technical Systems Framework. AMIA Annu. Symp. Proc..

[34] Burdine J.T., Thorne S., Sandhu G. (2020). Interpretive description: A flexible qualitative methodology for medical education research. Med. Educ..

[35] Thorne S. (2016). Interpretive Description: Qualitative Research for Applied Practice.

[36] (2023). ATLAS.ti Scientific Software Development GmbH. ATLAS.ti Windows (Version 23.2.3), ATLAS.ti Web (Version 5.11.1). https://atlasti.com.

[37] Giacomini M.K., Cook D.J. (2000). Users’ guides to the medical literature: XXIII. Qualitative research in health care A. Are the results of the study valid? Evidence-Based Medicine Working Group. JAMA.

[38] Parker M.G., Garg A., McConnell M.A. (2020). Addressing Childhood Poverty in Pediatric Clinical Settings. JAMA Pediatr..

[39] Horbar J.D., Edwards E.M., Ogbolu Y. (2020). Our Responsibility to Follow Through for NICU Infants and Their Families. Pediatrics.

[40] Moreyra A., Dowtin L.L., Ocampo M., Perez E., Borkovi T.C., Wharton E., Simon S., Armer E.G., Shaw R.J. (2020). Implementing a standardized screening protocol for parental depression, anxiety, and PTSD symptoms in the Neonatal Intensive Care Unit. Early Hum. Dev..

[41] Constant B., Cullen D., Dalembert G., Duffy J., McPeak K., Scribano P., Vasan A., Wilson-Hall L. (2021). Screening for Social Needs in Pediatrics: How Can We Ensure It Is Family-Centered and Effective?. https://policylab.chop.edu/sites/default/files/pdf/publications/PolicyLab-Issue-Brief-Screening-For-Social-Needs-In-Pediatrics.pdf.

[42] Gottlieb L.M., Tirozzi K.J., Manchanda R., Burns A.R., Sandel M.T. (2015). Moving electronic medical records upstream: Incorporating social determinants of health. Am. J. Prev. Med..

[43] Pew Research Center (2021). Demographics of Mobile Device Ownership and Adoption in the United States. https://www.pewresearch.org/internet/fact-sheet/mobile/.

[44] Lorch S.A., Rogowski J., Profit J., Phibbs C.S. (2021). Access to Risk-Appropriate Hospital Care and Disparities in Neonatal Outcomes in Racial/Ethnic Groups and Rural-Urban Populations. Semin. Perinatol..

